# Draft Genome Sequence of *Bacillus amyloliquefaciens* AP183 with Antibacterial Activity against Methicillin-Resistant *Staphylococcus aureus*

**DOI:** 10.1128/genomeA.00162-15

**Published:** 2015-04-16

**Authors:** Shamima Nasrin, Mohammad J. Hossain, Mark R. Liles

**Affiliations:** Department of Biological Sciences, Auburn University, Auburn, Alabama, USA

## Abstract

*Bacillus amyloliquefaciens* AP183 expresses secondary metabolites that inhibit the growth of methicillin-resistant *Staphylococcus aureus* (MRSA). Here, we present a ~3.99-Mbp draft genome sequence of AP183 with the aims of providing insights into the genomic basis of its antibacterial mechanisms and exploring its potential use in preventing MRSA skin colonization.

## GENOME ANNOUNCEMENT

Strains within the *Bacillus subtilis* group, which includes the species *Bacillus amyloliquefaciens*, are known to produce industrially important enzymes and bioactive compounds and have been used as biocontrol agents against plant and animal pathogens (1–3). Strain AP183 is a plant growth-promoting rhizobacterium (PGPR) isolated from a cotton plant rhizosphere and was found to belong to *B. amyloliquefaciens* subsp. *plantarum*, based on a phylogenetic analysis of the *gyrB* and 16S rRNA gene sequences.

Strain AP183 genomic DNA was extracted (4), and a bar-coded library was constructed using a Nextera kit. Genome sequences were generated on an Illumina MiSeq sequencer using a 2 × 250 paired-end sequencing kit. The sequence reads were trimmed for quality and assembled using the CLC Genomics Workbench (CLC bio, Cambridge, MA), obtaining 1,331,792 sequence reads, with an average coverage of 36×. The sequence reads were assembled *de novo*, generating 40 contigs >500 bp in length, with an *N*_50_ of 190,739 bp, and the largest contig was 541,177 bp. The estimated genome size was ~3.99 Mbp, with an average G+C% of 46.4%, which is very similar to that of other *B. amyloliquefaciens* genomes (5). Gene prediction and protein annotation were performed using the RAST server (6). A total of 4,005 open reading frames (ORF) were predicted, of which 74% had a significant BLAST hit (*E* value, <0.001), and 41 tRNA genes were predicted.

Secondary metabolite biosynthesis gene clusters were predicted using anti-SMASH2.0 (7), which resulted in 18 predicted secondary metabolite biosynthesis gene clusters containing 566 genes. AP183 is predicted to encode five *trans*-acyltransferase (AT) polyketide synthases (PKS), three nonribosomal peptide synthetases (NRPS), two hybrid PKS-NRPS, one hybrid *trans*-AT PKS, one type I PKS, one type II PKS, one type III PKS, and two terpene and two bacteriocin biosynthesis gene clusters. The AP183 genome is also predicted to contain a cluster with ORFs with homology to genes in the bacilysin biosynthetic cluster (8). In addition, an NRPS biosynthetic gene cluster was predicted in the AP183 genome with no known homology to that of other *Bacillus* species but with homologs within the genome of *Cyanothece* sp. strain PCC 7424. Recently, a novel antibacterial compound, bacillusin A, was discovered from AP183 with potent activity against methicillin-resistant *Staphylococcus aureus* (MRSA) and other bacterial pathogens (9). Based on its structure, the predicted biosynthetic gene cluster responsible for bacillusin A synthesis is a *trans*-AT PKS pathway.

We found that the genome of AP183 contains two genes predicted to encode resistance to the antibiotics fosfomycin and fluoroquinolone, but no genes predicted to encode virulence factors were identified within this genome. The AP183 genome sequence will contribute to future studies to characterize the secondary metabolite biosynthetic pathways involved in anti-MRSA activity and to determine the safety of this strain in inhibiting MRSA colonization.

### Nucleotide sequence accession numbers. 

The draft genome of AP183 has been deposited as a whole-genome shotgun sequencing project at DDBJ/EMBL/GenBank under the accession no. JXAM00000000. The version of strain described in this paper is the first version, JXAM01000000.
